# Uncertainty analysis for an effluent trading system in a typical nonpoint-sources-polluted watershed

**DOI:** 10.1038/srep29398

**Published:** 2016-07-11

**Authors:** Lei Chen, Zhaoxing Han, Guobo Wang, Zhenyao Shen

**Affiliations:** 1 State Key Laboratory of Water Environment, School of Environment, Beijing Normal University, Beijing 100875, P.R. China; 2 Transport Planning and Research Institute Ministry of Transport of the People’s Republic of China, P.R. China

## Abstract

Conventional effluent trading systems (ETSs) between point sources (PSs) and nonpoint sources (NPSs) are often unreliable because of the uncertain characteristics of NPSs. In this study, a new framework was established for PS-NPS ETSs, and a comprehensive analysis was conducted by quantifying the impacts of the uncertainties associated with the water assimilative capacity (WAC), NPS emissions, and measurement effectiveness. On the basis of these results, the uncertain characteristics of NPSs would result in a less cost-effective PS-NPS ETS during most hydrological periods, and there exists a clear transition occurs from the WAC constraint to the water quality constraint if these stochastic factors are considered. Specifically, the emission uncertainty had a greater impact on PSs, but an increase in the emission or abatement uncertainty caused the abatement efforts to shift from NPSs toward PSs. Moreover, the error transitivity from the WAC to conventional ETS approaches is more obvious than that to the WEFZ-based ETS. When NPSs emissions are relatively high, structural BMPs should be considered for trading, and vice versa. These results are critical to understand the impacts of uncertainty on the functionality of PS-NPS ETSs and to provide a trade-off between the confidence level and abatement efforts.

As a particular application of market principles, effluent trading systems (ETSs) allow the cost-effective abatement of specific pollutant loadings on water bodies at the watershed scale^1^. Recently, costly technologies have been required to meet the limits of effluents from point sources (PSs), so the inclusion of nonpoint sources (NPSs) in conventional ETSs has become important^2^. First, NPSs account for the majority of the total pollutant emissions in many watersheds, so the regulation of NPSs would lead to greater gains in emission control^3^. Second, NPSs are generally low-cost dischargers when site-specific best management practices (BMPs) are implemented^4^. Third, compared to the conventional command-and-control method, PS-NPS ETSs might be more effective at regulating water quality than PS ETSs because farmers are not responsible for controlling pollutant-enriched runoff^5^.

However, PS-NPS ETSs have not been implemented successfully, especially in terms of the number and type of trading participants^6^,^7^. PS-NPS ETSs have problematic aspects, mainly because of the specific characteristics of NPSs. First, NPSs are driven by random weather-related forcing variables, i.e., rainfall. Owing to their inherently stochastic nature, NPS emissions can be neither defined nor treated as a constant, in contrast to PSs that have clearly known discharge streams. Typically, NPS emissions are expressed in terms of the expected emission loading instead of the emission variability, so deterministic PS emissions would be traded with stochastic (uncertain) NPS emissions^8^. Second, effluent permits are created based on the water assimilative capacity (WAC) of the receiving water body. In conventional ETSs, the dry-season WAC is often applied as the worst case scenario, or the most vulnerable condition, to provide a safety margin^9^. However, this is not the case for those typical NPS-polluted rivers, in which the WAC changes significantly over time owing to the variation of flow and the physical–chemical–biological processes that occur within the river system^10^. Third, abatement efforts for NPSs are often simulated mathematically^11^,^12^. However, the effectiveness of BMPs is also uncertain owing to imperfect knowledge and limited experience^13^. In this way, a failure to characterize these routine uncertainties prohibits the achievement of water quality goals and increases the risk posed by PS-NPS ETSs for each regulated river.

Because those NPSs are not perfect substitutes for PSs, a considerable number of studies have focused on the stochastic nature of NPS emissions in order to generate a reliable ETS. By tracking the uncertainty of the driving factors, various WACs, including time-varying, flow-variable, and weighted sum permits, have been proposed^14^. NPS emissions have also been treated as specific probability distributions around the expected discharge loads^2^,^15^,^16^. In addition, the uncertainty ratio has been introduced to quantify the minimum level of NPS emissions that is required to offset a unit of the PS load^17^. In this way, a PS-NPS ETS would depend on the relative marginal abatement costs and uncertainties associated with flow and pollutant loadings would be considered^4^,^14^. Uncertain parameters, which are often drawn from reports or the literature, have been specified for ETS models. Horan *et al*.^18^ addressed the uncertain parameters in an ETS by an ex post Monte Carlo analysis. Li *et al*.^19^ developed a recourse-based interval fuzzy programming approach by incorporating interval, fuzzy and probabilistic forms into an ETS. Zhang *et al*.^16^ advanced a two-stage stochastic program for agricultural ETSs in which each uncertain parameter was treated as a probability distribution. However, there are still concerns about PS-NPS ETSs that do not adequately consider all of the uncertain characteristics of NPSs^2^.

This research attempts to fill this scientific gap. First, a new ETS framework is proposed by incorporating the uncertainties associated with the WAC, NPS emissions, and BMP effectiveness. Second, the impacts of these uncertainties, and the choice of hydrological period and BMP type on the trading results of a PS-NPS ETS are quantified. This new framework is then applied to the control of total phosphorus (TP) in a typical NPS polluted river in China.

## Materials and Methodology

### Study area

**Figure 1:**
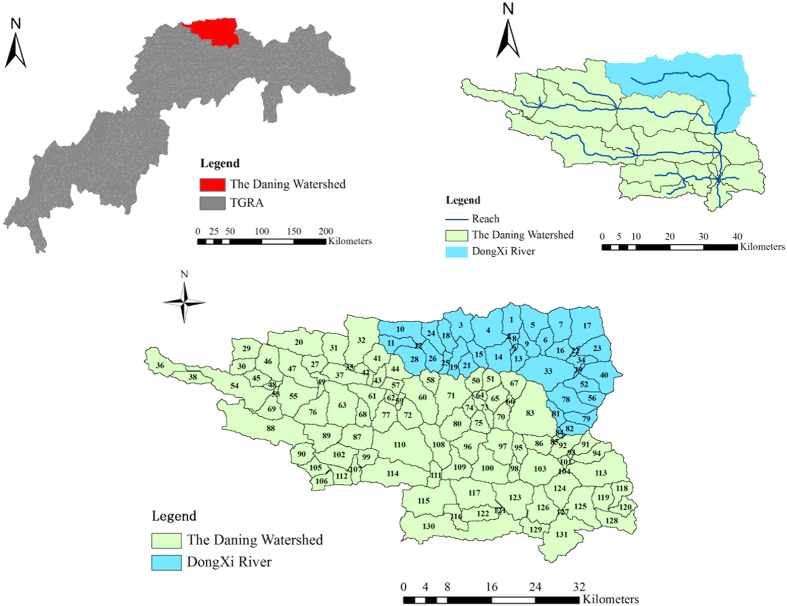
The location and formation of the Dongxi River watershed. This figure was created by the Arcmap software, which can be downloaded from the website of http://www.arcgis.com/features/.

The Daning River watershed, located in the center of the Three Gorges Reservoir Area, was selected as the study area (Fig. 1). Detailed meteorological, topographical, land use and soil data on this watershed can be found in our previous studies^10^,^20^. To simplify the ETS market, the Dongxi River, which is a key tributary of the Daning River and also a whole water environmental functional zone (WEFZ), was selected. This river flows through the northwestern mountainous areas to the terrain of the southern plain and covers a drainage area of 572.06 km^2^. In this region, agricultural activity represents the major land use, and only two PSs, Xujiazhen and Bailuzhen, were available as appropriate candidates for trading. Compared to these PSs, NPS pollution is increasingly severe owing to the high local population density and intensive agricultural activities. Specifically, high TP loadings from NPSs have periodically resulted in algal blooms and local eutrophication at the downstream WEFZ boundary of the Dongxi River, which is the chief river section of concern for water quality purposes. On the basis of the national standard (GB3838-2002), the Dongxi River is regarded as a habitat for rare aquatic beings, and it also provides drinking water to local residents. According to this designated use (class II WEFZ), this downstream boundary was selected as the targeted assessment section, and its TP standard was set as 1.0 mg/L.

As illustrated in Fig. 1, the whole Dongxi River watershed was delineated as a fully distributed river system that consists of almost 37 small sub-watersheds. This formation provides a fine resolution similar to the spatial requirement of the structural BMPs to be built. The PS emissions were calculated using discharge reports and measured TP concentration data, which were provided by local waste water treatment plants. Available GIS data, such as land use data, digit elevation models, and soil maps, were used to generate land surface characteristics within each spatial unit. Government information and site surveys were also used to identify the attribute data, i.e., the fertilizer amount, of local NPS dischargers within the watershed.

In this study, the selection of BMPs stemmed directly from our discussions with local watershed managers and farmers. Currently, nutrient management (the reduction of the local nutrient amount), which is a representative type of non-structural BMP, has already been implemented widely in this region. Structural BMPs have not yet been required but are of great interest to local managers. In this research, the detention pond, as an artificially constructed pond or tank, was also selected to reduce NPS pollutants through its gravitational settling and other bio-chemical mechanisms.

### Methodology

To incorporate trading uncertainty, the new PS-NPS ETS consists of a) a Monte Carlo-based WAC calculation, b) a chance constraint-based method for treating NPS emissions, c) a watershed model for quantifying the uncertain abatements of NPSs emissions, d) simple cost equations for assessing economic decisions, and e) a linear programming-based algorithm for optimizing trade results.

### The Monte Carlo-based WAC calculation

Generally, the WAC represents the ability of the receiving water bodies to assimilate certain targeted pollutants without exceeding the water quality standard^21^. The whole year was divided into three hydrological seasons based on local historical records; June, July, and August were defined as the wet season; January, February, March, November and December as the dry season; and April, May, September, and October as the normal season^22^. Here, a classic one-dimensional WAC model was used to quantify the maximum number of loading permits for a specific pollutant that can be emitted into the targeted water body but can still maintain the TP concentration under 1.00 mg/L.


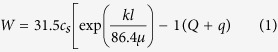


where *W* is the WAC of the targeted river body (ton); *q* and *Q* represent the effluent amount of the discharge and the upstream river flow, respectively (m^3^/s); *c*_*s*_ is the TP standard (mg/L); *k* represents the transfer coefficient of TP (s^−1^); and *l* and *u* are the river length (km) and the velocity of water (m^3^/s), respectively.

In eq. (1), the flow rate (*Q*) and transfer coefficient (*u*) bear the largest portion of uncertainties so these two parameters were specified a priori^14^. Owing the limitation of the data, the flow at the downstream boundary of the Dongxi River was calculated by the SWAT model, which was developed by Arnold *et al*.^23^ and has been used widely in this region^10^,^20^. The time series of simulated daily flows are then specified probabilistically instead of based on conventional determinative values^18^. In this research, the flow data during a hydrological season were observed to follow the characteristics of a normal distribution, which is consistent with previous studies^24^,^25^. Specifically, the expected value and standard deviation of the flow data were calculated to be 2.52, 13.93, and 26.20 m^3^/s and 2.74, 10.45, and 18.09 m^3^/s for the wet, normal and dry season, respectively. Moreover, the transfer coefficient is defined as the fraction of pollution emission that would be delivered to the targeted river section^1^,^9^,^26^. For most areas, this specified coefficient is drawn from related studies or reports, which might cover a range of values. For simplicity, the delivery coefficient was assumed to follow a uniform distribution with a range of 0.019—0.062 according to the bounds suggested by previous studies^18^,^27^.

The Monte Carlo (MC) method was then selected to generate the probability distributions of seasonal WACs because of its simple concept, sound theory, and flexible usage. The MC method represents, thus far, the most powerful and compatible tool for modeling stochastic WACs, which is a typical nonlinear and complex problem^18^. In this research, three MC procedures were conducted: (1) a set of flow rate and transfer coefficient values was sampled from their probability distributions by using random numbers; (2) all random combinations of these two parameters were input into the WAC model, and eq. (1) was run; and (3) the statistical characteristics of WAC outputs were analyzed to generate their cumulative probability distributions^28^. The prior analysis for the distribution of flow data was performed using Microsoft Excel 2013^29^, and MC modeling was conducted by using the EPA-FYNTOX software program, the concept of which is described in Marr and Canale^30^. Finally, a total of 10,000 simulations were performed by random sampling of independently distributed input parameters.

### The chance constrained programming-based NPS emission

In the second step, NPS emissions were quantified at the sub-watershed scale using the SWAT model. The good-of-fit indicators, in terms of Nash-Sutcliffe coefficients, were calculated as 0.89 and 0.66 for flow simulation and 0.75 and 0.46 for TP prediction during the calibration and validation periods, respectively, which indicated that the SWAT performs well in this region. More details about the model evaluation can be found in our previous studies^20^,^31^. Owing to the random nature of the forcing factors, the NPSs emissions during a certain period could be characterized as stochastic variables, with their mean value and the variance of the loadings shown in Table 1 8. In this way, the use of an expected value and its variance for NPS-TP loadings can be a substitute for NPS emission uncertainty, especially when many interlinked factors must be considered and related information is often scarce.

The TP concentration at a specific river section can be calculated as a function of effluents (from both PSs and NPSs), and the natural degradation process of TP along the river. Based on our previous work^22^, a classic one-dimensional water quality model was used to quantify the water quality response under specific emission conditions.


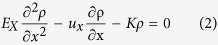


The solution of *p* and *R*_*d*_ for a typical degradable pollutant can be expressed as:


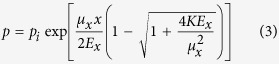


where *p*_*i*_ and *p* represent the TP emission from discharger *i* and its remaining amount at the targeted river section, respectively (tons); *x* is the river length from discharger *i* to the targeted river section (m); *u*_*x*_represents the average flow (m/d or m/s); and *E*_*x*_ and K are the vertical mixing coefficient (m^2^/d or m^2^/s) and degradable coefficient of P (1/d or 1/s), respectively.

Compared to PSs, which discharge from explicit outlets, NPSs are often assumed to be located evenly along the main body of the targeted river. Thus, the NPS loadings were assumed to be emitted into the targeted water body in an even manner. The NPSs-TP loadings per reach length are expressed as 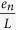, in which *e*_*n*_ and *L* represent the total NPS-TP emission and the river length, respectively. If the deliver function of P and its remaining load are assumed to be *u(x)*, and 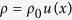, the integral expression can be expressed as 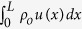 and its solution of the functional area at (*0*, *L*) is:


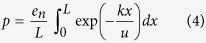


At this step, the problem becomes how the random variable *e*_*n*_ affects optimal trading results. One convenient approach for solving this stochastic problem is to use the chance constrained programming approach developed by Charnes and Cooper^32^. In this approach, the probabilistic constraint of NPS emissions is replaced by its deterministic equivalent. Assuming that the NPS emissions are normally distributed, the deterministic equivalent of *e*_*n*_ can be written as:


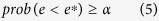


The solution of eq. (5) is:


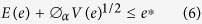


where e^*^ represents the stochastic PS-NPS emissions and the required WAC obtained from the cumulative distribution function, and *E*(*e*) and *V*(*e*) represent the expected data and variance, respectively, of pollutant loads during a certain hydrological period.

### The uncertainty of NPS abatement

In the third step, we focused on the uncertainty of NPS abatement. Generally, the location and type of BMPs represent the most important variables for NPS control^33^,^34^. The selection of BMPs is typically based on multiple factors, including site characteristics, i.e., slope, soil infiltration, or water table elevation, and other considerations such as land use regulation^35^. Considering these constraints, it is impossible to implement BMPs in every candidate location within a watershed. Thus, a topography analysis was performed first by evaluating the surface status, slope and land use type at the sub-watershed scale^36^.

The SWAT model was then used to assess the abatement efforts of BMPs. Using the crop-growth module, nutrient reduction is simulated by changing the agricultural management operations, i.e., the schedule and amount of fertilizer^37^. Conversely, the detention pond is delineated by modifying specific parameters that influence watershed hydrology and nutrient cycles via structural BMPs. Typically, the coefficient of permeability (K), which represents the permeable bottom of the land surface, is used to delineate the infiltration process and subsequently the water storage of the detention pond^38^. In this research, the value of K was set as 1.0 mm/h if the detention pond was chosen for a specific sub-watershed. Finally, a new matrix *y* = (*y*_1_, …, *y*_*i*_, …, *y*_*n*_) was developed to represent the effectiveness of BMPs, and *e*′ = *e* − *y* represents the TP emissions during the BMPs scenarios.

However, it is challenging to precisely define the effectiveness of BMPs (*y*) because there is no explicit process in the SWAT representation, and the NPS abatement itself might be stochastic^12^. In this way, an uncertainty ratio *ε* was introduced to represent the stochastic events that influence the effectiveness of BMPs, which could be characterized as:


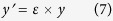


where *y′* and *y* represent the stochastic and baseline effectiveness of BMPs, respectively, and *ε* is the uncertainty ratio; if *ε* is set as 0, the stochastic NPS abatement transforms into the baseline deterministic form.

The NPS emissions at the sub-watershed scale under the BMP scenarios (assuming BMPs are placed at the sub-watershed scale) can then be expressed as:


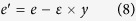


After incorporating the uncertain efficiency of BMPs, the water quality response at the downstream WEFZ boundary was calculated using eq. (3). Generally, the stochastic variable (*ε*) can be obtained from the long-term statistics of monitoring reports or case-by-case examination^11^. Because of imperfect knowledge, the value of *ε* was assumed to be 0.7, 0.8, 0.9, 1.0 and 1.1 in this research, depending in part on the reliability of the SWAT model.

### The cost function

The fourth step focuses on the estimation of the potential cost savings that the PS-NPS ETS program can provide. The equations described by Wang *et al*.^39^, which were derived from collected monitoring and related cost reports, were used as the basic abatement cost functions for PSs. Based on local investigations, the annual capital and operation/maintenance costs were 1.10 and 0.27 (10^6^¥), respectively, for the waste-water treatment plant of Bailuzhen and Xujiazhen, and their treatment levels were 10,000 and 3,000 m^3^/L*day. For PSs, the baseline TP loadings were calculated based on the collected discharge flow and permitted TP concentration. To maintain simplicity, we did not formally consider transaction expenditures (in terms of exchange costs and monitoring costs). The abatement cost function of each PS was then fitted as:


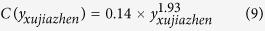



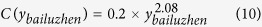


where *y*_*xujiazhen*_ and *y*_*bailuzhen*_ represents the abatement load of each PS, in terms of Xujiazhen and Bailuzhen, respectively.

The abatement cost for non-structural BMPs is typically a function of farmer decisions involving land use, labor, and fertilizer^27^. In this research, the abatement cost for nutrient reduction is related to the amount of fertilizer, and this basic information was obtained from our investigations with local farmers. These cost abatement functions were then used in the following optimization analysis:


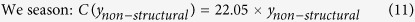



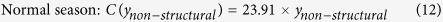



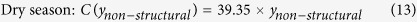


where *y*_*non−structural*_ represents the abatement loads of TP by non-structural BMPs.

**Figure 2:**
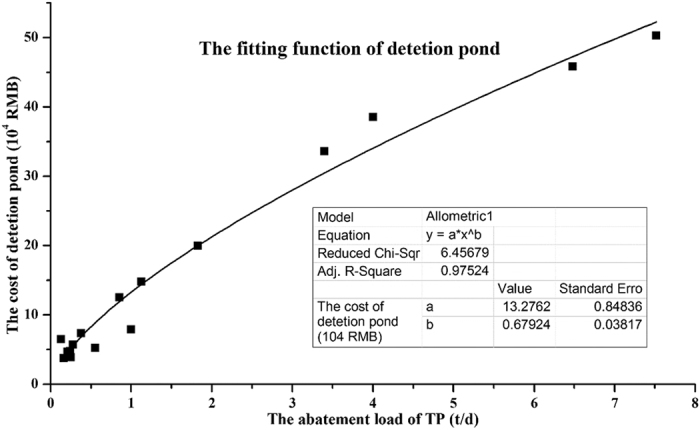
The fitted abatement cost function of detention pond.

With the aid of the SWAT model, the abatement load of the detention pond was generated, and the related cost data were obtained from the BMP database^40^. Based on local characteristics, the capacity of the detention pond was designed to provide a 1-day hydraulic retention time for local runoff at the sub-watershed scale^36^, whereas the flow and pollutant emissions during the BMP scenarios were generated by running the SWAT model. A conventional least-squares approach was applied to generate the required regression equations^14^. The scatter plots in Fig. 2 represent the abatement load and cost of the detention pond and the abatement cost function was fitted with respect to R^2^ criteria as 0.97.


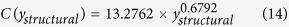


where *y*_*structural*_ represents the abatement TP loads by the structural BMPs.

### The linear programming-based optimization function

To determine the potential for PS-NPS trading, a linear programming-based algorithm was applied to quantify the abatement loads and related cost for PSs and NPSs. In this research, the linear-programming optimization algorithm was written using the General Algebraic Modeling System^14^,^41^. The targeted objective was to minimize the total abatement cost, and the constraint conditions were revised to incorporate the uncertainties related to the WAC, NPS emission, and NPS abatement. The final trading results provide the optimal allocation of TP credits among polluters and the related abatement cost while satisfying the probabilistic WAC constraint.


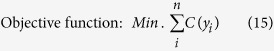



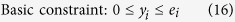



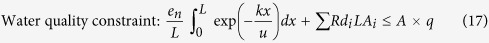



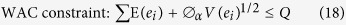


where *e*_*i*_ and *y*_*i*_are the baseline emission and its abatement load, respectively; *c(y*_*i*_) represents the abatement cost (¥); and *A* and *q* are the TP standard (mg/L) and design flow at the downstream boundary (m^3^/s).

## Results

### The impacts of WAC uncertainty

**Figure 3:**
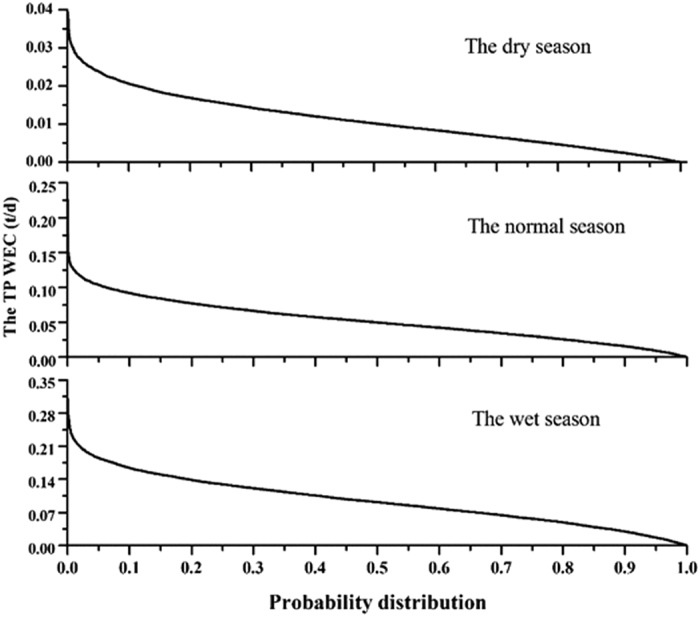
The cumulative distribution curves of TP-WACs during different seasons.

The cumulative distribution curves of the WACs outputs are presented in Fig. 3, in which the positive and negative values represent seasonal WACs that can be allocated and the conditions in which the TP exceeds the standard at the downstream WEFZ boundary, respectively. Moreover, each point in the cumulative curve represents a certain WAC value according to different assumptions of the flow and the transfer coefficient. As illustrated in Fig. 3, the variability in the WACs is significant with wide ranges of 0 to 0.39, 0 to 0.22, and 0 to 0.04 ton/day for the wet, normal, and dry seasons, respectively. The WAC value clearly decreases with increasing cumulative probability owing to the higher frequency of the requirement for meeting the TP standard, which indicates that the choice of a proper WAC should be a trade-off between the permitted amount of initial discharge and the confidence requirement. In this way, a conventional fixed WAC, which could be obtained by assuming Q90, Q75 or 7Q10 flow conditions^42^, is inappropriate for the Daning River (a typical NPS-polluted river).

**Figure 4:**
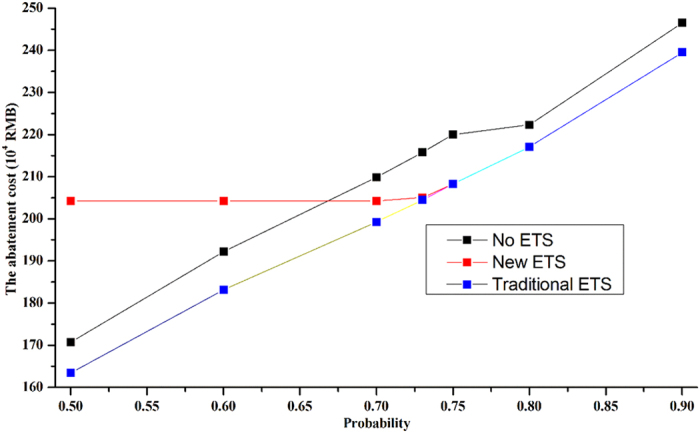
The impacts of WAC uncertainty on the trading results of different ETSs.

In this section, 7 confidence levels (50%, 60%, 70%, 72%, 75%, 80% and 90% cumulative probability) were addressed to quantify the impacts of WAC uncertainty on the trading results of the PS-NPS ETS. For comparison, three schemes were designed: a WEFZ-based ETS (in which both the downstream water quality constraint and the WAC constraint should be satisfied); a conventional ETS (in which only the WAC constraint is considered); and a no trading condition (in which initial permission for the TP load is allocated proportionally among all of the sources based on their current loadings) (Han *et al*.^22^). As shown in Fig. 4, the WAC uncertainty shows greater impacts on the trading results of the conventional ETS and the no trading condition, whereas the total abatement cost increases gradually with increasing confidence level. When the cumulative probability increases from 50% to 90%, the abatement expenditure at the optimal trade equilibrium increases by 46.62% and 46.47%, respectively, for these two methods, which indicates that the imposition of the higher confidence level requirements is costly. In comparison, if the confidence level is higher than 72%, the WAC uncertainty shows similar impacts on the trading results of the WEFZ-based and conventional ETSs, but little impact on the WEFZ-based ETS could be observed under this specific confidence level. As the confidence level decreases from 90% to 50%, only a 17.15% cost savings can be observed using the WEFZ-based ETS, and the difference in the abatement cost between the WEFZ-based and conventional ETSs becomes gradually larger. Under the confidence level of 67%, the WEFZ-based ETS is no longer cost effective compared to the baseline case of no trading. This was because as the confidence level increases, a transition from the water quality constraint to the WAC constraint for the PS-NPS ETS essentially exists.

Similar impacts could also be observed on the total abatement load and the optimal allocation of credits among the sources. When the confidence level changed from 50% to 90%, the total abatement load increased by 48.32%, 48.32% and 17.87% for the no trading condition, conventional ETS, and WEFZ-based ETS, respectively. The variability of the confidence level also leads to different water quality responses. For example, if the conventional ETS was used, the downstream TP concentration was less than 0.1 mg/L during all hydrological seasons when the confidence level was set as 75%. However, if the confidence level was 73%, the TP concentration was more than 0.1 mg/L during the normal and dry seasons. Furthermore, if the confidence level decreased to 70%, the water quality could not meet the standard requirements during any of all hydrological seasons. In comparison, using the WEFZ-based ETS, achievement of water quality had no direct relationship with variability in the confidence level but did reduce the cost effectiveness of the ETS market.

### Impact of hydrological period

In this section, we focus on the choice of the hydrological period, which is critical for designing a proper ETS^4^. Figure 3 implies that the WAC curves exhibit temporal variability, the values of which are set as follows: wet period > normal period > dry period. For example, if the confidence level was set as 70%, the seasonal TP-WAC was quantified as 15.64, 11.34 and 2.71 tons for each hydrological period, respectively. However, this ranking is different from that in a previous study, which demonstrated that the normal-season WAC is higher than that of dry and wet periods in the Baixi River watershed^43^. This is because the Baixi River is affected by the regulation of a nearby reservoir, whereas the Dongxi River, which is located in the central part of Three Gorges Reservoir Area, consists mainly of natural rivers. Thus, the variability in the seasonal WAC in the Dongxi River watershed corresponded to the natural hydrological periods, whereas precipitation in the wet season would increase the storage volumes and carrying capacity of the pollutants^26^. In comparison, owing to the regulation of the Baixi Reservoir, the discharge flow was low in the wet season to control the flood pulse, which resulted in a smaller WAC than in the other two seasons.

The PS-TP and NPS-TP emissions for the study area are presented in Table 1. Compared to the PSs, the NPSs-TP loadings are higher, which indicated that there is the potential for a PS-NPS ETS in this watershed. As shown in Table 1, the NPSs emissions varied significantly among the hydrological periods, in which the expected load and variance of NPSs emissions ranged from 648 to 34,487 kg and from 358 to 40,903 kg, respectively. Based on the year scale, there is clearly an increasing trend in NPS emissions from the dry period to the wet period: i.e., the variance in the wet-season was 1,362, 5,985, and 9,311 kg for dry, normal, and wet years, respectively. However, within a specific hydrological year, larger variances might be observed during the normal season because more months (April, May, September, and October) are included in this period than in the wet season. Specifically, the highest and lowest uncertainties related to NPS emissions could be observed in the normal (season)-normal (year) and dry-dry period, respectively.

The trading results of the PS-NPS EST during the different hydrological periods were then quantified and compared. For simplicity, the confidence level of the seasonal WAC was set as 70%, and only the WEFZ-based ETS framework was considered. As shown in Table 2, the choice of the hydrological period has great impacts on the trading results. In total, the optimal abatement load and related cost at the optimal trading equilibrium ranged from 1,931 kg to 124,572 kg and from 4.74*10^4^ ¥ to 276.29*10^4^ ¥, respectively. Moreover, the allocation of credits among PSs and NPSs varies according to the choice of the hydrological periods. During the wet-dry, normal-dry and wet-normal periods, the WAC constraint shows greater impact, and the trading among PSs and NPSs is based on their marginal abatement cost. In comparison, because of the smaller flow rate and pollutant carrying capacity, the water quality constraint becomes more important in the dry-dry period, so the marginal abatement cost of PSs and NPSs become unequal^2^. However, because those two PSs are close in location, they showed similar impacts on the downstream water quality; thus, PS-PS trading is still in accordance with the principle of marginal abatement cost.

During the normal- and dry-normal periods, the variance of NPSs emissions is greater than that of river flow, so the water quality constraint has a greater impacts on the trading results. Instead of their marginal abatement cost, the credits would be allocated optimally between sources based on their contributions to downstream water quality, which could be defined as the relative delivery of TP loading to a shared critical location^21^. During a wet year, owing to the larger amount of rainfall and NPS emissions, the abatement load and cost changed from 28,433 kg to 109,519 kg, and from 599*10^4^ ¥ to 2,430*10^4^ ¥, respectively. Specifically, the required abatement load exceeded the expected value of TP emission during the wet-wet period, which indicates that the WAC constraint cannot be satisfied even when most NPS emissions are removed. Thus, further reduction of the PS loads is required, resulting in a high abatement cost in the wet-wet period.

### The impact of NPS emission variability

In eq. (6), *V*(*e*) and *ϕ*_α_ represent the variance of NPS emissions and the standard normal value, respectively, under the specific confidence level *α*. For simplicity, in above sections, no emission variability is assumed by setting the value of *α* and *ϕ*_α_ as 0.5 and 0, respectively. In this section, to quantify the impacts of emission variability, four scenarios were further designed by setting the value of *α* as 0.6, 0.65, 0.7 and 0.75. As shown in Table 3, the emission variability showed little impact on the trading results of PSs in most hydrological periods except for the wet-wet period, in which the abatement load and abatement cost of PSs increased sharply by 42.70% and 61.74%, respectively, from the no emission scenario. This can be explained by Table 2; the WAC constraint is difficult to satisfy in this period even though most NPS emissions are removed, so further reduction of PSs is required.

In comparison, the variability in NPS emissions had a large impact on the abatement efforts for NPSs in most hydrological periods (expect for the wet-wet, normal-normal, dry-normal, and normal-wet periods). As illustrated in Table 3, when the value of *α* increased from 0.6 to 0.75, the abatement loads of NPSs increase by 20.70% and 21.60% during the normal-dry and dry-dry periods, respectively. However, a change of only 0.05% could be observed for those two PSs. Moreover, the ratio of the abatement load (*y*) to the emission variance (*V*(*e*)) increased from 12 to 15. These impacts were amplified even during the wet-normal, wet-wet and dry-wet periods, with the increase in the abatement load by 149.08%, 37.47% and 31.01%, respectively. This demonstrated that the increase in the emission variability would lead to less cost-effective PS-NPS ETS markets.

Table 3 also shows that the emission variability has significant influence on the allocation of credits between PSs and NPSs. An increase in emission variability would cause the abatement efforts to shift from NPSs toward PSs. The rule of thumb is that if the variability in the NPS emissions is quite large, stricter regulations on PSs are needed, which would increase the abatement cost or decrease the market flexibility^1^. Moreover, when the NPS’s variability is large, the water quality constraint becomes more important compared to the WAC constraint. Thus, the credit (abatement load) would be allocated between sources based on their contributions to downstream water quality. To make the PS-NPS ETS more attractive, the confidence level of the WAC should be adjusted to cover the variability in the NPSs emissions. Another consideration might be to use a reserve ratio or margin of safety for a reliable ETS, in which some of the total TP permits are treated as an additional insurance WAC by quantifying the variability in NPS emissions^7^.

### Impact of abatement uncertainty

Generally, the type and location of BMPs are two important decision variables for NPSs abatement^33^,^34^. For simplicity, only non-structural BMPs, in terms of nutrient management, are considered in the above analyses. In this section, structural BMPs (the detention pond scenario) are further simulated and compared. Specifically, the total abatement cost is calculated by summing the construction cost of the detention pond at each sub-watershed, which was closely related to the maximum NPS emission during a specific hydrological period^38^. As shown in Table 4, the allocation of the abatement load and cost in the detention pond scenario was similar to that of the non-structural BMPs. However, the detention pond would result in less abatement loadings when the NPS emission is relatively low, which is different from the non-structural BMPs. This difference would be amplified during the wet-dry and dry-dry periods, in which the NPS emissions were only 8,917 and 3,244 kg, respectively. This is because the marginal abatement cost of the detention pond is higher during these periods, so the regulation of PSs would be preferred, resulting in a less cost-effective market. However, when NPS emissions are relatively high during wet-wet or other periods, the number of detention ponds increases significantly, and the marginal abatement cost would decrease accordingly, which illustrates the economic advantage of NPS abatement^44^. These results indicate that ‘amount effects’ of structural BMPs exist, which induce more cost-effective PS-NPS ETS when NPS emission is relatively high and vice versa^34^,^38^.

In the above analysis, abatement uncertainty was neglected by assigning the uncertainty factor (a) a value of 1.0. In this section, the uncertainty factor (a) was further set as 0.7, 0.8, 0.9 and 1.1 to quantify the uncertainties of the BMPs’ effectiveness, in which 0.7 indicates that the reduction of 1 unit of an NPS load would result in only a 70% actual abatement of NPS emission. As shown in Table 5, as the value of *α* decreased from 1.0 to 0.7, the respective abatement loads during the wet, normal, and dry years increased by 40.99%, 53.21%, and 31.13% for NPSs and 59.90%, 59.85%, and 59.80% for PSs, respectively. This indicates that the increase in abatement uncertainty will definitely reduce the credits of NPSs at the optimal trading equilibrium. In this way, the uncertainty associated with BMP effectiveness should be identified as a barrier to NPS-PS trading programs. However, if the uncertainty factor is set as 1.1, a decreasing trend in the total abatement load could be observed, indicating a more cost-effective ETS market. These results illustrate the importance of reporting the uncertainties related to BMP effectiveness, which was also highlighted by Arabi *et al*.^45^. Moreover, the decreased uncertainty factor would result in an increasing marginal abatement cost for both PSs and NPSs. Specifically, when there is no abatement uncertainty, the WAC constraint becomes more important and the marginal abatement costs of NPSs and PSs are equal. As illustrated in Table 5, when the uncertainty factor was increased to 1.1, the changes in the marginal abatement costs of NPSs was higher, indicating that more abatement efforts would come from the regulation of NPSs. This result demonstrates that the increase of BMPs’ effectiveness would reduce the marginal cost of NPS credits and makes them more attractive than those of PSs^7^.

When developing a PS-NPS ETS, watershed managers often requires PSs to purchase credits from NPSs at a trading ratio of 4:1 or 2:1, which is related to the uncertainty of BMP efficiency and NPS emission variability^14^,^46^. However, determining a proper ratio is a complex task, and a case-by-case uncertainty analysis is needed^4^. On the basis of this research, we suggest that the uncertainty ratio should be used to specify the actual emission abatement during the BMP scenario, whereas the marginal abatement cost of NPSs and PSs provides a practical substitute for the conventional trading ratio. For example, if the uncertainty factor is set as 0.7, 0.8, 0.9 or 1.1, the ratio of the marginal cost of PSs to that of NPSs is calculated as 1.4, 1.2, 1.1 and 0.9, respectively, which represents the reduction of NPSs emissions necessary to offset a 1-unit increase in PSs emissions^1^,^41^. Another effective way for the PS-NPS ETS to work might be the use of more effective models or more scientific research on BMP efficiency^7^.

### Implications

In this research, a new framework was established for a reliable PS-NPS ETS, and a comprehensive analysis was conducted to quantify the impacts of the trading uncertainties. On the basis of the results, the uncertainty had a great impact on the trading results, and the design of the PS-NPS ETS would involve a trade-off between the confidence level and abatement efforts. Moreover, the uncertainty showed less impact on the trading results of PSs in most cases, but the larger uncertainty in NPSs emission and abatement would definitely cause the abatement efforts to shift from NPSs toward PSs. Specifically, WAC uncertainties showed less impacts on the WEFZ-based ETS, but as the confidence level increased, a transition from the water quality constraint to the WAC constraint occurred. When the NPS emission is relatively high, structural BMPs should be considered for a more cost-effective ETS, and vice versa. In general, this new framework provides practical ways to efficiently incorporate NPS-based uncertainty into the existing PS-NPS ETS structure.

However, because PS-NPS ETSs have not been widely implemented, more preliminary research is needed. First, farmers are not responsible for controlling agricultural NPSs. Thus, the choice of an appropriate permit baseline is difficult for those NPSs. Second, many interlinked factors exist, so the design of a PS-NPS ETS and the regulation and monitoring of NPSs should be conducted with care under uncertain conditions, especially for NPSs-polluted river watersheds. Third, although this research highlights the use of the uncertainty ratio, reserve ratio and effective model, related information is often scarce, so more case-by-case studies are required.

## Additional Information

**How to cite this article**: Chen, L. *et al*. Uncertainty analysis for an effluent trading system in a typical nonpoint-sources-polluted watershed. *Sci. Rep.* **6**, 29398; doi: 10.1038/srep29398 (2016).

## Figures and Tables

**Table 1:** The effluent loads of PSs and NPSs and their impacts on water quality of the Dongxi River Watershed.

Period		Dry year			Normal Year			Wet Year		
Source		Wet season	Normal Season	Dry Season	Wet season	Normal Season	Dry Season	Wet season	Normal Season	Dry Season
NPS (kg)	Emission amount	8917	14572	3244	14969	137948	14320	27201	136884	34035
	Expected value	2970	3643	648	4989	34487	2864	9067	34221	6807
	Standard Deviation	1362	3530	358	5985	40903	5760	9311	37238	13245
PS (kg)	Xujiazhen	1375	1833	2291	1375	1833	2291	1375	1833	2291
	Bailuzhen	325	433	541	325	433	541	325	433	541
TP concentration (mg/L)		0.086	0.27	0.21	0.13	0.90	0.63	0.16	0.47	0.59
Achieve standard?		No	Yes	Yes	Yes	Yes	Yes	Yes	Yes	Yes

**Table 2:** The abatement load and cost of the WEFZ-based ETS in different hydrological periods.

Period		Dry year		Normal year			Wet year			
Source		load (kg)	Cost (10^4^ ¥)	Marginal cost (¥)	load (kg)	Cost (10^4^ ¥)	Marginal cost (¥)	load (kg)	Cost (10^4^ ¥)	Marginal cost (¥)
Wet season	Xujiazhen	340	1.08	22.05	340	1.08	22.05	946	7.76	56.97
	Bailuzhen	118	0.41	22.05	118	0.41	22.05	285	2.56	56.97
	NPS	1472	3.25	22.05	13247	29.21	22.05	27201	59.98	22.05
	Total	1931	4.74	—	13707	30.7	—	28433	70.30	—
Normal season	Xujiazhen	501	2.28	24.15	489	2.18	23.63	485	2.14	23.45
	Bailuzhen	171	0.89	24.15	171	0.89	24.06	170	0.87	23.88
	NPS	14572	32.13	23.91	1223911	273.22	23.91	108863	240.04	23.91
	Total	15246	35.30	—	124572	276.29	—	109519	243.06	—
Dry season	Xujiazhen	1503	18.97	54.5	1134	11.01	41.94	1149	11.3	42.46
	Bailuzhen	456	6.8	54.5	364	4.25	42.71	362	4.21	42.46
	NPS	3244	7.15	39.35	12853	28.34	39.35	34035	75.05	39.35
	Total	5204	32.93	—	14352	43.61	—	35548	90.56	—

**Table 3:** The impact of NPSs emission uncertainty on the trading results of the WEFZ-based ETS.

Confident level	Hydrological season	Dry year				Normal year				Wet year			
		Load (kg)		Cost (10^4^¥)		Load (kg)		Cost (10^4^¥)		Load (kg)		Cost (10^4^¥)	
		PS	NPS	PS	NPS	PS	NPS	PS	NPS	PS	NPS	PS	NPS
0.6	Wet	461	1472	1.51	3.25	459	5059	1.49	11.17	459	19786	1.49	43.63
	Normal	674	11838	3.18	28.31	661	123911	3.06	296.27	655	117745	3.02	281.53
	Dry	1401	2601	13.39	10.24	1498	13721	15.27	53.99	1397	6777	13.28	26.67
0.65	Wet	461	1472	1.51	3.25	459	7394	1.49	16.30	459	23417	1.49	51.64
	Normal	674	11838	3.18	28.31	661	123911	3.06	296.27	655	117745	3.02	281.53
	Dry	1397	2643	13.28	10.4	1498	13721	15.27	53.99	1397	7427	13.28	29.23
0.7	Wet	461	1472	1.51	3.25	459	9908	1.49	21.85	585	27201	2.41	59.98
	Normal	670	12170	3.14	29.1	661	123911	3.06	296.27	655	117745	3.02	281.53
	Dry	1397	2894	13.28	11.39	1498	13721	15.27	53.99	1397	8128.75	13.28	31.99
0.75	Wet	461	1472	1.51	3.25	459	12601	1.49	27.79	1699	27201	13.92	59.98
	Normal	666	14289	3.11	34.17	661	123911	3.06	296.27	655	117745	3.02	281.53
	Dry	1397	3163	13.28	12.45	1498	13721	15.27	53.99	1397	8879	13.28	34.94

**Table 4:** The abatement load and related cost in the detention pond scenario.

Hydrological period		Abatement load (kg)		Cost (10^4^ RMB)	
Year	Season	PS	NPS	PS	NPS
Dry	Wet	849.99	230.16	19.32	0.1
	Normal	1755.52	10744.88	20.74	0.1
	Dry	2833.33	1167.2	52.49	0.1
Normal	Wet	1428.6	6184.05	13.79	0.1
	Normal	707.2	128554.28	3.50	0.1
	Dry	2362.05	12797.95	37.14	0.1
Wet	Wet	1037.1	15003.66	7.38	0.1
	Normal	715.72	127088.88	3.57	0.1
	Dry	1578.45	32572.8	16.87	0.73

**Table 5:** The impact of abatement uncertainty on the trading results.

Period	Abatement load (kg)	0.7	0.8	0.9	1	1.1
Wet year	PSs	662	576	510	457	414
	NPSs	21966	19327	17253	15580	15580
	The rate of cost	1.4	1.2	1.1	1	0.9
Normal year	PSs	665	579	512	459	416
	NPSs	13860	12235	10950	9908	9046
	The rate of cost	1.4	1.2	1.1	1.0	0.9
Dry year	PSs	668	581	514	461	418
	NPSs	1807	1689	1576	1472	1378
	The rate of cost	1.4	1.3	1.1	1.0	0.9
